# Josephson Diode Effect in Parallel-Coupled Double-Quantum Dots Connected to Unalike Majorana Nanowires

**DOI:** 10.3390/nano14151251

**Published:** 2024-07-25

**Authors:** Yu-Mei Gao, Hu Xiao, Mou-Hua Jiang, Feng Chi, Zi-Chuan Yi, Li-Ming Liu

**Affiliations:** 1 School of Electronic and Information Engineering, UEST of China, Zhongshan Institute, Zhongshan 528400, China; yumeigao@zsc.edu.cn (Y.-M.G.); yizichuan@zsc.edu.cn (Z.-C.Y.); liulmxps@zsc.edu.cn (L.-M.L.); 2 Zhongshan Zhuoman Microelectronics Co., Ltd., Zhongshan 528400, China; hu.xiao02@zsmls.com; 3 South China Academy of Advanced Optoelectronics, South China Normal University, Guangzhou 510006, China; 2022024132@m.scnu.edu.cn

**Keywords:** Josephson diode effect, double-quantum dots, diode efficiency, quantum interference effect, Majorana bound states

## Abstract

We study theoretically the Josephson diode effect (JDE) when realized in a system composed of parallel-coupled double-quantum dots (DQDs) sandwiched between two semiconductor nanowires deposited on an s-wave superconductor surface. Due to the combined effects of proximity-induced superconductivity, strong Rashba spin–orbit interaction, and the Zeeman splitting inside the nanowires, a pair of Majorana bound states (MBSs) may possibly emerge at opposite ends of each nanowire. Different phase factors arising from the superconductor substrate can be generated in the coupling amplitudes between the DQDs and MBSs prepared at the left and right nanowires, and this will result in the Josephson current. We find that the critical Josephson currents in positive and negative directions are different from each other in amplitude within an oscillation period with respect to the magnetic flux penetrating through the system, a phenomenon known as the JDE. It arises from the quantum interference effect in this double-path device, and it can hardly occur in the system of one QD coupled to MBSs. Our results also show that the diode efficiency can reach up to 50%, but this depends on the overlap amplitude between the MBSs, as well as the energy levels of the DQDs adjustable by gate voltages. The present model is realizable within current nanofabrication technologies and may find practical use in the interdisciplinary field of Majorana and Josephson physics.

## 1. Introduction

The diode is one of the basic devices in semiconductor technology, and it is characterized by a high asymmetry of resistance in opposite directions. It is one of the central building blocks required in the fields of computation and electronic devices. In many superconductor-based systems, a similar effect named the superconductor or Josephson diode effect (JDE) appears due to broken time-reversal and inversion symmetries [1,2,3,4,5,6]. The JDE can result in direction-dependent (nonreciprocal) critical currents driven by the phase difference between superconductors, and it is fascinating in various applications due to its connection to the fundamental properties of diverse superconducting systems [1,2,3,4,5,6,7,8]. Generally, time-reversal symmetry may be broken by an external magnetic field, which exerts impacts on the specific term in the systems’ Hamiltonian related to inversion symmetry breaking [9], such as the Rahsba spin–orbit interaction (RSOI). Therefore, the JDE can be used to detect RSOI strength [10] or the existence of a topological phase in the nanowires that are proximity-contacted with a superconductor [5,10,11]. The JDE is also promising in the design of electronic devices including photodetectors, ac/dc converters, superconducting qubits [8], racetrack memory devices [12], etc.

The JDE was demonstrated as early as the 1970s in superconducting quantum interference devices (SQUIDs) based on superconductor bridges [13] and Josephson junctions [14]. It was also observed in non-centrosymmetric conventional superconductor thin films in terms of device geometry [15]. Since then, many experimental and theoretical platforms for the realization of JDE have been continuously proposed, such as non-centrosymmetric superconductors [14,15], stacks of different superconductors with broken inversion symmetries [16], Andreev molecules [17], artificial superlattices [2], topological semimetals and insulators [5], insulator heterostructure devices [18], nanowires [10], and disordered systems [19]. Another efficient platform proposed to achieve high diode efficiency relies on a Josephson current interferometer, in which conjunct Josephson junctions with nonsinusoidal current-phase relations form a SQUID. In such systems, the Josephson currents are contributed from higher harmonics other than the usual 2π-periodic ones, with a magnetic flux penetrating through the SQUID loop [6,8,14]. It has recently been demonstrated in two-dimensional electrons and many three- and four-terminal setups, in which the diode efficiencies at equilibrium can reach to up to about 30% [20,21].

Conventional s-wave superconductors that are proximity-contacted with semiconductor nanowires, which have strong RSOI and are subjected to external magnetic fields, have been demonstrated for the realization of topological superconductivity [6,8,10,11]: a topological state that hosts Majorana bound states (MBSs) [22,23]. It is well known that electrons are solutions to the Dirac equation, which is a complex relativistic quantum mechanical equation for particles obeying fermionic statistics. In 1937, however, Ettore Majorana from Italy presented a real equation for relativistic quantum fermions [24]. Particles satisfying Majorana equations are correspondingly named Majorana fermions. Due to the realness of the equation, Majorana fermions are their own antiparticle and have Hermitian creation and annihilation operators. In recent years, Majorana fermions have been prepared in solid-state platforms as charge-neutral topologically protected quasiparticles, i.e., MBSs [25]. They offer an attractive way for constituting Majorana qubits [26,27], allowing one to store information in a nonlocal manner that is immune to decoherence by a local disturbance [28]. Along with the intensive investigations on preparation and detection of MBSs, exploiting their possible applications is also an active research subject in condensed matter physics. For example, in systems with MBSs that are side-coupled to quantum dots (QDs), which are connected to external leads, the sign of thermopower that measures the induced bias voltage in response to a temperature difference can be reversed by QD-MBS hybridization strength or MBS–MBS overlap amplitude [29,30]. Moreover, the magnitudes of both the thermopower and thermoelectric efficiency in such systems can be noticeably enhanced [29,30,31]. Since the MBSs often emerge with the help of spin–orbit interactions, strong magnetic fields, or magnetic materials, they also play an important role in the research field of spintronics [32]. In recent years, some works have been devoted to the study of Josephson currents through a QD connected to two semiconductor nanowires hosting MBSs (MNWs) [33,34,35,36,37], which are stimulated by the interesting results found in various systems with QDs connected to superconductors with an ordinary phase [38,39,40,41,42,43,44,45,46]. It has been shown that a Josephson current driven by topological phase difference is quite a bit stronger than that driven by an ordinary phase difference, and the bent angle formed by the two MNWs, as well as the magnetic fields in the QD, will significantly suppress the Josephson current [33,34].

**Figure 1:**
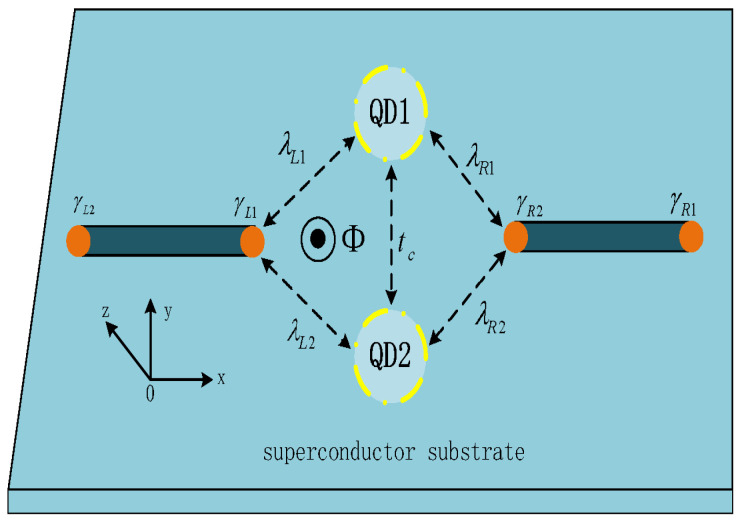
Schematic diagram for the studied system, which is composed of parallel double-quantum dots (DQDs) coupled to the left and right nanowires hosting Majorana bound states (MBSs) at their ends. The MBSs are denoted by $\gamma_{\alpha i}$ with α=L,R and i=1,2, and they interact with the QDs with strengths of $\lambda_{\alpha i}$. The DQDs are coupled to each other via a tunnel barrier of amplitude $t_{c}$. In the presence of a magnetic flux Φ threading through the system, a phase factor ϕ was added to $\lambda_{\alpha i}$ in addition to the phase difference from the superconductor substrate $\varphi_{\alpha }$. The relationship between $\lambda_{\alpha i}$ and the phase factors ϕ and $\varphi_{\alpha }$ will be specified in the main text.

Until now, there have been two limitations in previous works on the Josephson current through MNW–QD–MNW systems: one is that only the current’s magnitude and period were studied, and less attention was paid to the control of its direction [33,34,35,36,37]; the other is that only a single QD was inserted between two MNWs, and the interesting quantum interference effect was left untouched [14,16,20,21,39,41,42,43,44,47,48]. In view of these, we propose a structure composed of parallel double QDs (DQDs) sandwiched between the left and right MNWs to achieve the JDE based on the quantum interference effect arising from the two transport paths through the DQDs, as well as the magnetic flux penetrating through the loop, which is shown in Figure 1. We emphasize that the present device can be experimentally realized by, for example, combing the two similar systems reported in Refs. [45,49,50]. In Refs. [45,50], parallel DQDs were successfully inserted between two conventional superconductors, which may be further driven into a topological superconductor state hosting MBSs [26] and then becomes the present device. In Ref. [50], serial DQDs connected to two normal leads were coupled to each other via both a superconductor hosting MBSs and a normal tunnel barrier. In some previous works, DQDs have been inserted between conventional superconductor leads to generate spin-correlated electron pairs, as well as to control the Josephson current and its critical one [41,42,44]. Our studies show that, in this MNW–DQD–MNW system, the period, magnitude, and the directions of the Josephson current can be effectively adjusted with the help of dot energy levels, overlap amplitude between the MBSs, as well as the magnetic flux through the loop. Accordingly, a tunable JDE with a large value of diode efficiency emerges, and it may find real use in the design of superconductor-based instruments.

## 2. Model and Methods

The Hamiltonian of the present structure was divided into three parts as $H=H_{DQDs}+H_{MNWs}+H_{T}$ [33,34,51,52], in which the Hamiltonian of the DQDs and interaction between them is given by

(1) $$\begin{array}{c} H_{DQDs}=\sum_{i}\varepsilon_{i}d_{i}^{†}d_{i}+t_{c}\left(d_{1}^{†}d_{2}+d_{2}^{†}d_{1}\right), \end{array}$$

where the creation (annihilation) operator $d_{i}^{†}$$\left(d_{i}\right)$ is for electrons in dot-*i* with a spin-independent energy level $\varepsilon_{i}$. In experiments, the dot levels can be tuned via gate voltages $V_{g}$, and they are given by $\varepsilon_{i}=\varepsilon_{i}^{0}-eV_{g}$, where $\varepsilon_{i}^{0}$ is the bare energy level in dot-*i*. Here, we adopted a spinless model due to the helical property of MBSs, which may couple only to a unique spin state on the QDs [25,28,51,52]. The tunnel coupling strength between the DQDs is $t_{c}$. The Hamiltonian $H_{MNWs}$ denotes the left and right MNWs connected to the DQDs, whose explicit expression is as follows [33,51,52]:

(2) $$\begin{array}{c} H_{MNWs}=i\sum_{\alpha =L,R}\varepsilon_{\alpha }\gamma_{\alpha 1}\gamma_{\alpha 2}, \end{array}$$

where $\varepsilon_{L/R}$ is the direct hybridization strength between the MBSs prepared at the ends of the α-th nanowire. In what follows, we assumed $\varepsilon_{L}=\varepsilon_{R}=\delta_{M}$. The creation and annihilation operators of the MBSs satisfied the relationship of $\gamma_{\alpha j}=\gamma_{\alpha j}^{†}\left(j=1,2\right)$ and $\left\{\gamma_{\alpha i},\gamma_{\alpha^{\prime }j}\right\}=2\delta_{\alpha ,\alpha^{\prime }}\delta_{i,j}$ [51,52] due to the unique self-conjugate character of the MBSs. Tunnel coupling between the DQDs and the MNWs was described by the Hamiltonian of $H_{T}=\sum_{\alpha =L,R}H_{D\alpha }$, in which [33]

(3a) $$\begin{array}{ccc} & & H_{DL}=\sum_{i=1,2}\left(\lambda_{Li}d_{i}-\lambda_{Li}^{*}d_{i}^{†}\right)\gamma_{L1}, \end{array}$$

(3b) $$\begin{array}{ccc} & & H_{DR}=i\sum_{i=1,2}\left(\lambda_{R}d_{i}-\lambda_{R}^{*}d_{i}^{†}\right)\gamma_{R2}, \end{array}$$

where $\lambda_{\alpha i}$ stands for the coupling strength between QD-*i* and the MNW-α. Note that there is a phase factor $\varphi_{\alpha }$ in $\lambda_{\alpha i}$, which arises from the proximity of the MNWs to the superconductor substrates and induces the Josephson current [1,4,6]. Furthermore, there was another phase factor in $\lambda_{\alpha i}$ due to the total magnetic flux $\Phi =\Phi_{L}+\Phi_{R}$, where $\Phi_{L/R}$ is the magnetic flux penetrating through the left/right subring. In the present paper, we assumed the magnetic fluxes of the two subrings to be the same, i.e., $\Phi_{L}=\Phi_{R}$, and $\phi =2\pi \Phi /\Phi_{0}$ with the flux quantum $\Phi_{0}=hc/e$ [41]. In combing the two phase factors $\varphi_{\alpha }$ and ϕ, the hybridization strengths between the DQDs and the MNWs are given by $\lambda_{L1}=\left|\lambda_{L1}\right|\exp\left(i\frac{\phi }{4}+i\varphi_{L}\right)$, $\lambda_{L2}=\left|\lambda_{L2}\right|\exp\left(-i\frac{\phi }{4}+i\varphi_{L}\right)$, $\lambda_{R1}=\left|\lambda_{R1}\right|\exp\left(-i\frac{\phi }{4}+i\varphi_{R}\right)$, and $\lambda_{R2}=\left|\lambda_{R2}\right|\exp\left(i\frac{\phi }{4}+i\varphi_{R}\right)$. In the present paper, we set $\varphi_{L}=\varphi$ and $\varphi_{R}=0$ for the sake of clarity. As usual, we made a unitary transformation to change the Majorana fermion representation to a conventional fermion representation one [51,52]: $f_{L/R}=\left(\gamma_{L/R1}+i\gamma_{L/R2}\right)/\sqrt{2}$ and $f_{L/R}^{†}=\left(\gamma_{L/R1}-i\gamma_{L/R2}\right)/\sqrt{2}$. Then, we rewrote the total Hamiltonian in a matrix form to calculate the Green’s functions needed for the Josephson current. On the basis of $\Psi ={\left(d_{1}^{†},d_{1},d_{2}^{†},d_{2},f_{L}^{†},f_{L},f_{R}^{†},f_{R}\right)}^{†}$, the transformed Hamiltonian $\tilde{H}=\frac{1}{2}\Psi^{†}H\Psi$ is given by [33,34,35]:

(4) $$\begin{array}{c} \tilde{H}=\left[\begin{array}{ccc} \;\;{\tilde{H}}_{DQDs} & {\tilde{H}}_{DL} & {\tilde{H}}_{DR}\\ \;\;{\tilde{H}}_{LD} & {\tilde{H}}_{L} & 0\\ \;\;{\tilde{H}}_{RD} & 0 & {\tilde{H}}_{R} \end{array}\right], \end{array}$$

in which the 4×4 sub-matrix ${\tilde{H}}_{DQDs}=\;$ diag $\left(\varepsilon_{1},-\varepsilon_{1},\varepsilon_{2},-\varepsilon_{2}\right)+t_{c}\sigma_{x}⊗\sigma_{z}$, with $\sigma_{x/z}$ being the Pauli matrix of the x/z-component, and the symbol ⊗ denotes the matrix direct product. Similarly, the 2×2 sub-matrix ${\tilde{H}}_{L}={\tilde{H}}_{R}=\delta_{M}\sigma_{z}$ and the interaction between the DQDs and MBSs are

(5a) $$\begin{array}{ccc} & & {\tilde{H}}_{LD}=\frac{1}{\sqrt{2}}\left[\begin{array}{cccc} \;\;-\lambda_{L1}^{*} & \lambda_{L1} & -\lambda_{L2}^{*} & \lambda_{L2}\\ \;\;-\lambda_{L1}^{*} & \lambda_{L1} & -\lambda_{L2}^{*} & \lambda_{L2} \end{array}\right], \end{array}$$

(5b) $$\begin{array}{ccc} & & {\tilde{H}}_{RD}=\frac{1}{\sqrt{2}}\left[\begin{array}{cccc} \;\;\lambda_{R1} & \lambda_{R1} & \lambda_{R2} & \lambda_{R2}\\ \;\;-\lambda_{R1} & -\lambda_{R1} & -\lambda_{R2} & -\lambda_{R2} \end{array}\right]. \end{array}$$

The dc Josephson current *J* tunneling between the two MNWs via the DQDs is calculated in terms of the non-equilibrium Green’s function technique [33,38,39,40,41,42]

(6) J=eh∫dεReTr[σ˜z(Σ˜aGda−Σ˜rGdr)]f(ε),

where ${\tilde{\sigma }}_{z}=1_{2\times 2}⊗\sigma_{z}$, and ${\tilde{\Sigma }}^{r/a}=\Sigma_{L}^{r/a}-\Sigma_{R}^{r/a}$ are with retarded/advanced self-energies due to the MNWs. The self-energies are calculated by $\Sigma_{\alpha }^{r/a}={\tilde{H}}_{D\alpha }g_{\alpha }^{r/a}{\tilde{H}}_{\alpha D}$ [33]. The free retarded/advanced Green’s function of the MNW is $g_{\alpha }^{r/a}={\left[\varepsilon 1_{2\times 2}-{\tilde{H}}_{\alpha }+\pm i0^{+}\right]}^{-1}$. The retarded/advanced Green’s function of the DQDs is then obtained with the help of Dyson’s equation as $G_{d}^{r/a}={\left[\varepsilon 1_{4\times 4}-{\tilde{H}}_{DQDs}-\left(\Sigma_{L}^{r/a}+\Sigma_{R}^{r/a}\right)\right]}^{-1}$ [33,38,39,40,41,42]. In Equation (6), $f\left(\varepsilon \right)=1/[1+\exp\left(\varepsilon /k_{B}T\right)]$ is the equilibrium Dirac–Fermi function, where *T* and $k_{B}$ denote the temperature and Boltzmann constant, respectively.

## 3. Numerical Results

**Figure 2:**
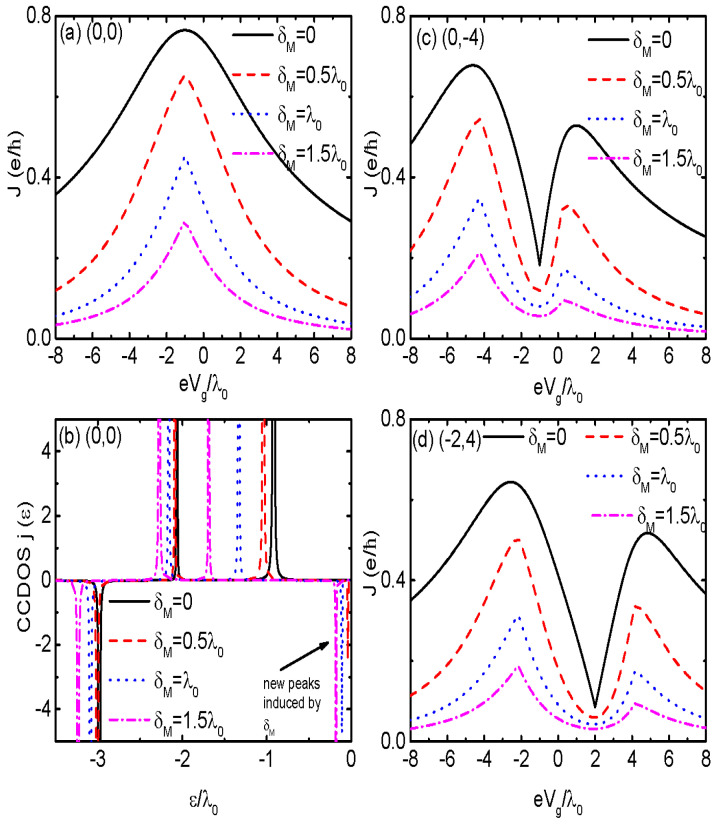
The (color online) Josephson current and CCDOS individually in (**a**,**b**) for the case of dot levels $\varepsilon_{1}^{0}=\varepsilon_{2}^{0}=0$, i.e., the configuration of (0, 0). (**c**) and (**d**) are for the Josephson current in the configurations of (0, −4) and (−2, 4), respectively. The tunnel-coupling strength between the dots were fixed at $t_{c}=\lambda_{0}$, ϕ=π, and φ=π/2.

In the numerical calculations, we considered the case of DQDs coupled to the left and right MNWs with equal strengths $|\lambda_{\alpha i}|=\lambda_{0}\equiv 1$, which were set to be the energy unit. In Figure 2, we present the gate voltage $V_{g}$ dependence of the Josephson current *J* with different direct hybridization coupling strength between the MBSs’ $\delta_{M}$ and energy levels $\left(\varepsilon_{1}^{0},\varepsilon_{2}^{0}\right)$ for a fixed ϕ=π and φ=π/2. First, we diagonalized the Hamiltonian $H_{DQDs}$ for the DQDs and transformed the QDs’ discrete energy states into a pair of bonding and antibonding states as follows:

(7) $$\begin{array}{c} H_{DQDs}=\varepsilon_{+}c_{+}^{†}c_{+}+\varepsilon_{-}c_{-}^{†}c_{-}, \end{array}$$

in which $c_{-(+)}$ is the annihilation operator for the bonding (antibonding) state of the DQDs with energy

(8) $$\begin{array}{c} \varepsilon_{\pm }=\bar{\varepsilon }\pm \sqrt{{\left(\frac{\Delta \varepsilon }{2}\right)}^{2}+t_{c}^{2}}, \end{array}$$

where $\bar{\varepsilon }=\left(\varepsilon_{1}+\varepsilon_{2}\right)/2$ and $\Delta \varepsilon =\varepsilon_{1}-\varepsilon_{2}$.

For the case of the QDs’ energy level configuration being (0,0) and $\delta_{M}=0$, there was only one current peak associated with the bonding state with energy $\varepsilon_{-}$. Therefore, the peak position will shift toward a lower (higher) energy regime with increasing (decreasing) $t_{c}$. Note, in this completely symmetrical structure, i.e., where the energy levels of the DQDs are the same (Δε=0) and are coupled to the MNWs with equal strengths $\left(|\lambda_{\alpha i}|=\lambda_{0}\right)$, the antibonding state $\varepsilon_{+}$ disappears and only the bonding state contributes to the Josephson current through the DQDs. When the MBSs at the opposite ends of the MNWs are hybridized to each other $\left(\delta_{M}\neq 0\right)$, the position of the Josephson current peak remains unchanged, as shown in Figure 2a. With increasing $\delta_{M}$, the current’s amplitude is reduced, which is consistent with previous results [33,34]. Moreover, the width of the peak is narrowed by increasing $\delta_{M}$. To explain the dependence of the Josephson current on the direct hybridization of the MBSs $\delta_{M}$, we showed the current-carrying density of states (CCDOS) j(ε)=ReTr[σ˜z(Σ˜aGda−Σ˜rGdr)]f(ε) varying with respect to energy ε. Since only the states located in a negative energy regime contribute to the Josephson current at zero temperature, we only showed the behaviors of j(ε) for the case of ε<0. When the MBSs at each MNWs were decoupled from each other $\left(\delta_{M}=0\right)$, there were three peaks in j(ε), of which the two positive and one negative are indicated by the black solid line in Figure 2b. When $\delta_{M}\neq 0$, a negative peak was induced in j(ε) around the Fermi energy of the MNWs μ=0, and its height was increased by increasing $\delta_{M}$. As a result of this, the current’s amplitude was reduced. The additional current peak originates from the fact that the MBSs in each MNWs are destroyed by their overlap [33,51,52].

If the energy levels in the two QDs are different from each other $\varepsilon_{1}\neq \varepsilon_{2}$, there are two current peaks corresponding to the two molecular states $\varepsilon_{\pm }$ in the DQDs, as shown in Figure 2c,d, in which $\left(\varepsilon_{1}^{0},\varepsilon_{2}^{0}\right)$ were set as (0, −4) and (−2, 4), respectively. The height of the current peak at bonding state $\varepsilon_{-}$ was lower than that at the antibonding state $\varepsilon_{+}$. When the MBSs in the same MNWs were overlapped ($\delta_{M}\neq 0$), the current peaks shown in Figure 2c,d were lowered and narrowed as in the case of Figure 2a. Moreover, the positions of the peaks were slightly changed by $\delta_{M}$ for the cases of $\varepsilon_{1}\neq \varepsilon_{2}$, which is different from the that of (0,0). It should be noted that the reduction in the Josephson current peaks in Figure 2c,d was mainly induced by the charge flowing through the two molecular states $\varepsilon_{\pm }$. This is different from the case in Figure 2a, where the charge flows only through the bonding state $\varepsilon_{1}$. This means that electrons will transport through the DQDs whenever their energy is in resonance with the bonding and antibonding states, even if $\varepsilon_{1}$ or $\varepsilon_{2}$ is away from the Fermi level.

**Figure 3:**
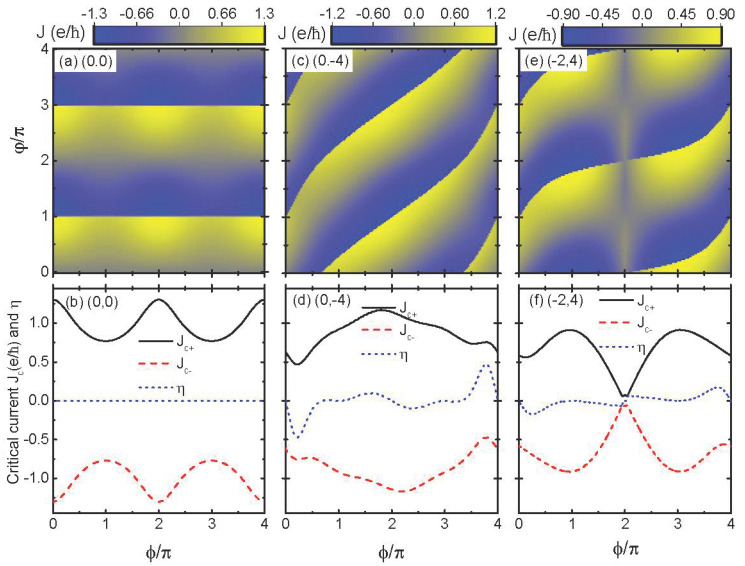
Josephson current *J* as a function of ϕ and φ, the positive (negative) critical current $J_{c+(-)}$, the diode efficiency η as functions of ϕ for the configurations of (0, 0) in (**a**) and (**b**), the (0, −4) in (**c**) and (**d**), and the (−2, 4) in (**e**) and (**f**) for φ=π/2, respectively. Other parameters are $t_{c}=\lambda_{0}$ and $eV_{g}=\delta_{M}=0$.

We then studied the influences of the magnetic flux ϕ and phase difference φ on *J*, the critical Josephson currents $J_{c\pm }$, as well as the diode efficiency $\eta =(J_{c+}-|J_{c-}|)/(J_{c+}+|J_{c-}\left|\right)$ [6,46] for different dot level configurations for a fixed value of $\delta_{M}=0$. For the case of (0,0), the oscillation period of both the Josephson current *J* and its critical counterpart $J_{c\pm }$ was 2π versus either ϕ or φ, as shown in Figure 3a,b. The Josephson current *J* in Figure 3a had an abrupt jump from positive to negative value at φ=nπ(n=0,1,2,⋯⋯). This is identical to the case in systems of a single QD sandwiched between two MNWs [33,34]. The current *J* in Figure 3a also had a maximum value at ϕ=nπ(n=0,1,2,⋯⋯), and it did not change its sign when ϕ was varied. This behavior was also identical to the structure in which the DQDs were connected to conventional non-topological superconductors (S-DQDs-S) [41]. The positive and negative critical currents as functions of ϕ in Figure 3b were antisymmetrical with respect to each other, i.e., $J_{c+}=-J_{c-}$; hence, the diode efficiency η≡0, which is indicated by the blue dotted line therein. For the cases of dot energy levels of (0,−4) and (−2,4), the Josephson current *J* in Figure 3c,e was a 4π-period function of ϕ and φ, and its sign depended on the values of both ϕ and φ. These results are similar to those found in S-DQDs-S [41]. This means that the normal 2π-period Aharonov–Bohm oscillations, such as for (0,0), was destroyed and complex periodic interference effects occurred by changing the dot levels [41]. Correspondingly, the electron transport processes including the current’s amplitude or directions are controllable by adjusting both the magnetic flux and the dot energy levels.

The period of the positive and negative critical currents $J_{c\pm }$ in Figure 3d,f was the same as that of *J*, which individually corresponded to the cases of (0, −4) and (−2, 4). Now, $J_{c+}$ and $J_{c-}$ are not antisymmetrical to each other, i.e., $J_{c+}\neq -J_{c-}$ at the greatest value of ϕ, and the phenomenon of JDE then emerged accordingly. We found that the diode efficiency was anti-symmetrical with respect to ϕ=2nπ and exhibited a triple-peak configuration, of which the two higher ones were located around ϕ=4nπ±π/4 and two lower ones at the two sides of ϕ=2nπ. This shows that η depends on the energy levels of the QDs and the magnetic flux. For example, the absolute maximum of η for (0,−4) in Figure 3d emerged at about 4nπ±π/4 and can reach up to 0.5, whereas for the level configuration of (−2,4), the absolute maximum of the diode efficiency was about 0.2 at almost the same value of ϕ. The above results show that the JDE can be efficiently controlled by the combined functions of QD energy levels and the magnetic flux that induces complex interference effects.

**Figure 4:**
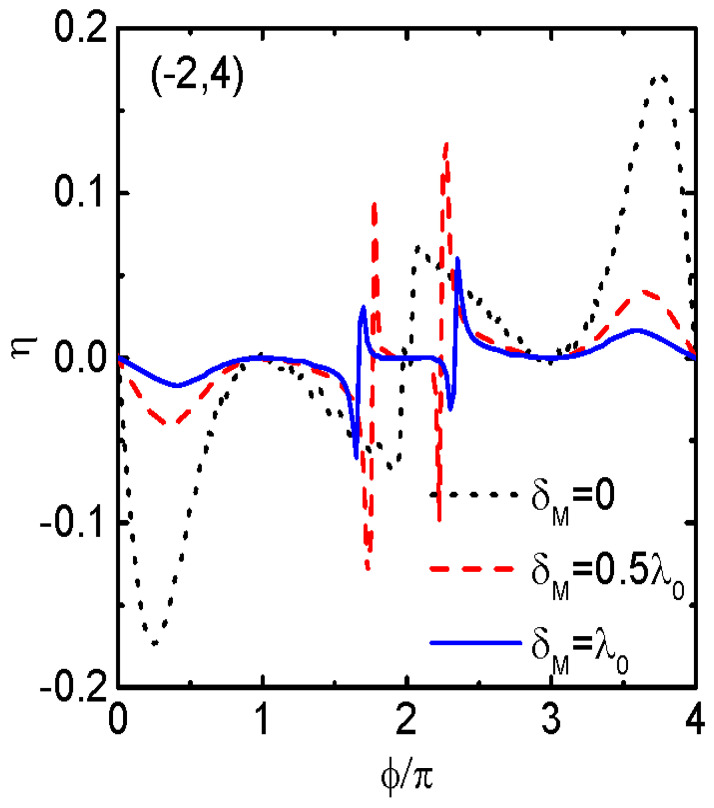
Diode efficiency η as a function of ϕ for the dot level configuration (−2,4) and different values of MBS–MBS overlap amplitude $\delta_{M}$ for φ=π/2. The other parameters are as in Figure 3.

In Figure 4, we present the impacts of the MBS–MBS overlap amplitude $\delta_{M}$ on the diode efficiency η with fixed QD-energy-level configurations (−2,4). From the figure, one can see that the diode efficiency remained as a 4π− periodic function of ϕ in the presence of finite $\delta_{M}$, and it is anti-symmetrical with respect to ϕ=2nπ. With increasing $\delta_{M}$, the peaks’ heights around ϕ=4nπ±π/4 were lowered with almost unchanged locations. The double-peak configuration around ϕ=2nπ in the case of $\delta_{M}=0$, however, evolved into a triple-peak one for $\delta_{M}\neq 0$, as is indicated by the red dashed and blue dotted lines. Moreover, the two pairs of peaks, which were shifted individually to magnetic flux values that were greater and smaller than ϕ=2nπ, kept the same shape as those of $\delta_{M}=0$, i.e., one positive and one negative. The heights of the two pairs of the peaks changed nonlinearly with respect to $\delta_{M}$. For the chosen values of $\delta_{M}$, the maximum of η first increased (by comparing the black dotted line for $\delta_{M}=0$ with the red dashed line for $\delta_{M}=0.5\lambda_{0}$) and then decreased (the blue solid line for $\delta_{M}=\lambda_{0}$). The results displayed in Figure 4 indicate that by properly adjusting the value of $\delta_{M}$, one can change either the amplitude or the sign of the diode efficiency. In the experiments, $\delta_{M}$ depended on the lengths of the MNWs and the superconductor coherence [25,26], which can all be used for changing the JDE.

**Figure 5:**
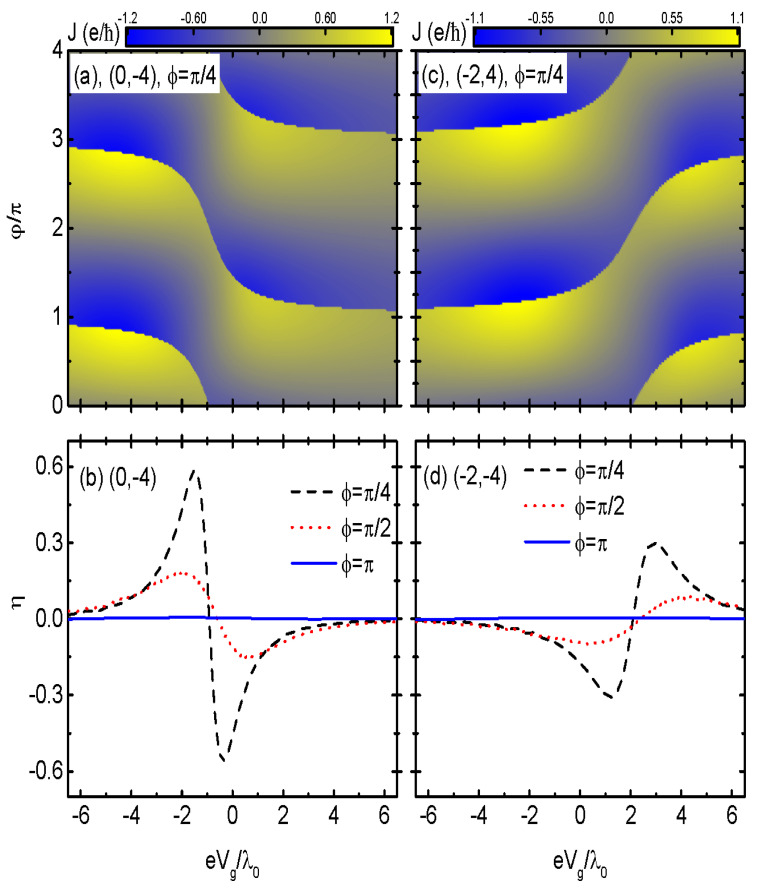
Josephson current *J* as a function of φ and $eV_{g}$ for ϕ=π/4, and the diode efficiency η as a function of $V_{g}$ for different values of ϕ. The dot level configurations are (0,−4) in (**a**) and (**b**), (−2, 4) in (**c**) and (**d**) at $\delta_{M}=0$, and φ=π/2 in (**b**) and (**d**). The other parameters are as in Figure 3.

Dependence of the Josephson current on the gate voltage $eV_{g}$ and phase difference φ for a fixed magnetic flux ϕ=π/4 and the different values of the bare dot energy levels are displayed in Figure 5a for (0,−4) and in Figure 5c for (−2,4). In both of the two cases of (0,−4) and (−2,4), the Josephson current with a varied function of $V_{g}$ was a 4π− periodic function of φ, and it jumped from positive to negative or vice versa at a particular φ. The value of the particular φ depends on both the dot energy level variance by the gate voltage $eV_{g}$ and the magnetic flux, whereas the amplitude of *J* had no obvious change. This result was quite different from that in S-DQDs-S, in which the current was significantly reduced as the dot energy levels were tuned away from the Fermi level [41]. As for the diode efficiency η, it was zero in the case of ϕ=π regardless of the value of dot energy levels $\varepsilon_{i}^{0}$, as is shown by the blue solid lines in Figure 5b,d. The diode efficiency η developed two peaks with opposite signs for the cases of ϕ=π/4 and π/2, and their locations and heights were varied when the value of the magnetic flux changed. Here, only the results of $\delta_{M}$ are displayed. We examined that, for a finite MBS–MBS overlap amplitude $\delta_{M}\neq 0$, the behaviors of the diode efficiency were similar to those shown in Figure 4, which are not listed here. It should be noted that although the present JDE emerged due to the phase difference between the left and right MNWs, it was observable by those means reviewed in Ref. [6] for the *orthodox* superconductor diode. This is because the MBSs were prepared on top of a superconductor substrate, and the phase factor added in the dot-MBS coupling strength arose essentially from the superconductivity, as those in Ref. [6]. The present paper shows that the existence of the MBSs will bring about some other interesting characteristics, as was compared to those previous JDEs.

## 4. Summary

In summary, we investigated the JDE in a MNW/DQD/MNW Josephson junction. It was found that when the energy levels of the DQDs are aligned with the Fermi levels in the MNWs, the Josephson current is a 2π-periodfunction with respect to both the phase difference arising from the superconductor substrate and the magnetic flux penetrating through the system. Now, the positive and negative critical Josephson currents were the same, and the JDE could not emerge. When the DQD energy levels were tuned away from the Fermi energies in the MNWs, the oscillation period of the Josephson current became 4π with respect to the magnetic flux, as well as the phase difference. Moreover, the positive and negative critical Josephson currents were different from each other and induced the JDE accordingly. Our results show that both the magnitude and the sign of the diode efficiency can be adjusted with the help of the DQDs’ energy levels and the overlap amplitude between the MBSs, as well as the value of the magnetic flux. The present results are beyond the reach of the MNW/singl-QD/MNW junction, and it can be realized with the help of current nanotechnology.

## Data Availability

All data included in this study are available upon request by contact with the corresponding authors.
